# The effect of induction method in twin pregnancies: a secondary analysis for the twin birth study

**DOI:** 10.1186/s12884-016-1201-8

**Published:** 2017-01-06

**Authors:** Elad Mei-Dan, Elizabeth V. Asztalos, Andrew R. Willan, Jon F. R. Barrett

**Affiliations:** 1 Women and Babies Program, Sunnybrook Health Sciences Center, Sunnybrook Research Institute, 2075 Bayview Ave, Toronto, ON M4N 3M5 Canada; 2 Child Health Evaluative Sciences, SickKids Research Institute, University of Toronto, The Hospital for Sick Children, 555 University Avenue, Toronto, M5G 1X8 ON Canada

**Keywords:** Cesarean section, Induction of labor, Prostaglandins, Twins

## Abstract

**Background:**

This secondary analysis for the Twin Birth Study, an international, multicenter trial, aimed to compare the cesarean section rates and safety between methods of induction of labor in twin pregnancies.

**Methods:**

Women with twin pregnancies where the first twin was in a cephalic presentation and who presented for labor induction, were non-randomly assigned to receive prostaglandin or amniotomy and/or oxytocin. Main outcome measures were the rates of unplanned cesarean section and neonatal and maternal mortality or serious morbidity.

**Results:**

153 (41.5%) were induced by prostaglandin (prostaglandin group) and 215 (58.5%) were induced by amniotomy and/or oxytocin alone (no prostaglandin group). Induction using prostaglandin was more common in countries with a low perinatal mortality rate <10/1000 (45.7 versus 32.5%, *p* = 0.02). Cesarean section rates were similar in the two groups: 62/153 (40.5%) in the prostaglandin group and 87/215 (40.5%) in the no prostaglandin group (odds ratio 1, 95% CI 0.65-1.5). Nulliparity, late maternal age, non-cephalic presentation of twin B and high country’s perinatal mortality rate were found to be independently associated with the induction to end with an unplanned cesarean section. There were no significant differences between groups with respect to maternal or neonatal adverse outcomes.

**Conclusions:**

The need for cervical ripening by prostaglandin had no effect on the incidence of cesarean delivery or an abnormal outcome. There is a significant risk of unplanned cesarean section independent of chosen induction method.

**Trial registration:**

This trial was registered at the International Standard Randomized Controlled Trial Register (identifier ISRCTN74420086; December 9, 2003) and retrospectively registered at the www.clinicaltrials.gov (identifier NCT 00187369; September 12, 2005).

**Electronic supplementary material:**

The online version of this article (doi:10.1186/s12884-016-1201-8) contains supplementary material, which is available to authorized users.

## Background

Twin birth are increasingly common and now occur in 2 to 3% of all births [1–3]. These pregnancies pose an increased risk for adverse perinatal outcomes compared to singletons at all gestations [1, 2]. In late twin pregnancy perinatal mortality rates rise dramatically from 8.4 per 1000 at 38 weeks of gestation to 12.7 and 15.6 per 1000 at 40 and 41 weeks, respectively [2, 4]. In attempt to limit these late term losses an elective delivery at 37–39 weeks of gestation has been widely recommended [1, 5–8]. This has led to a marked rise in induction of twin pregnancies from 5.8% in 1989 to 13.8% in 1999, with a concomitant decrease in twin stillbirth rates [9]. Oxytocin and prostaglandins (PG) are commonly used induction methods in twin gestations, however despite their increase use, information regarding the outcome of these induced labors is minimal [10–12].

The Twin Birth Study (TBS) was an international, multicenter, randomized controlled trial that compared planned vaginal delivery (VD) to planned cesarean section (CS) in twin pregnancies between 32 and 38 weeks gestation where the first twin was in a cephalic presentation at the time of randomization [1]. The study reported there was no difference in fetal and neonatal outcomes between the two approaches. In this nonrandomized secondary analysis of the TBS data on women who had induction of labor, the primary objective was to compare the rate of unplanned CS and safety between various induction methods that were employed both on neonatal and maternal mortality or serious morbidity.

## Methods

### Initial Study [1]

Women were enrolled in the TBS if they were between 32 and 38 weeks of gestation, the first twin was in the cephalic presentation, and both twins were alive with an estimated weight between 1500 g and 4000 g. Exclusion criteria were mono-amniotic twins, fetal reduction at 13 or more weeks of gestation, the presence of a lethal fetal anomaly, contraindication to labor or VD (e.g., fetal compromise, second twin substantially larger than the first twin, fetal anomaly or condition that might cause mechanical problems at delivery, and previous vertical uterine incision or more than one previous low-segment CS), and previous participation in the TBS. Participants aged under 16 years were not included in this study. Randomization took place between December 13, 2003 and April 4, 2011; women were randomly assigned to planned CS or planned VD. Randomization was centrally controlled at the Center for Mother, Infant, and Child Research at the Sunnybrook Health Sciences Center in Toronto, ON, Canada. Data were abstracted from the medical records at participating centers by trained study staff and were recorded, after delivery, on standardized data-collection forms. Participating centers were prepared to perform a CS within 30 min if necessary; and had anesthetic, obstetrical, and nursing staff available in the hospital at the time of delivery. A qualified obstetrician, experienced at vaginal twin delivery, was required to attend all vaginal deliveries. Elective delivery by means of either CS (for women in the planned CS group) or labor induction (for women in the planned VD group) was planned between 37 weeks 5 days and 38 weeks 6 days of gestation. The methods of induction, the use of oxytocin and the decision of oxytocin regimen were not prescriptive and each center/clinician could choose the preferred methods of induction and dosing. The patients included in the analysis for this paper were those who were randomized to planned VD and had labor induced.

Baseline factors, such as maternal age, parity, chorionicity, gestational age at induction and the country’s perinatal mortality rate (PMR), associated with the method of induction were compared with respect to discrete variables using contingency table chi-squared tests and with respect to continuous variables using two-sample t-tests. The effect of the method of induction on unplanned CS; neonatal mortality or serious neonatal morbidity; and, maternal death or serious maternal morbidity was investigated using logistic regression. The effect of the method of induction on the time intervals of the induction delivery process, viz., the duration between active labor and full dilation, the duration between active labor and delivery and the duration between delivery and discharge were investigated using two-sample t-tests. Odds ratios and their corresponding 95% confidence intervals are provided for binary outcomes, while means and standard deviations are provided for continuous outcomes. For the neonatal outcome the unit of analysis is infant and generalized estimating equations were used to account for the correlation between infants from the same pregnancy. All *p*-values in all tables are two-sided. The level for declaring statistical significance was set to 0.05. Since the analysis was secondary to a randomized clinical trial, no power calculations were performed. The power of the analysis and corresponding results are reflected in the width of the confidence intervals. This research adhered to the STROBE guidelines for observational studies.

## Results

**Fig. 1:**
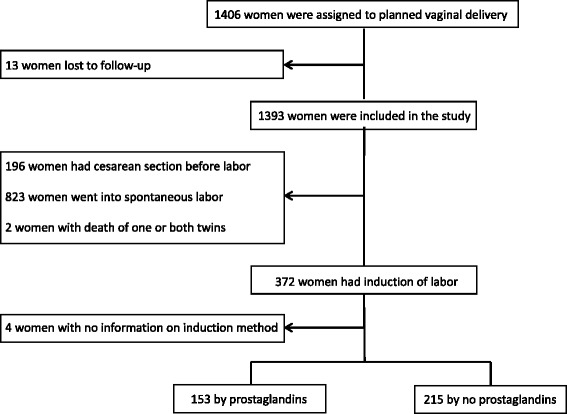
Flow diagram of women randomized to the planned vaginal birth arm of the Twin Birth Study

The planned VD arm was randomly assigned to 1406 women. Of these, 368 women were identified as having undergone induction: 153 (42%) underwent induction with the use of prostaglandin (PG group) and 215 (58%) underwent induction with amniotomy and/or oxytocin (no PG group), (Fig. 1). Table 1 outlines the characteristics of the women in the two comparison groups at the time of randomization and at the time of delivery. The two groups were similar apart from the national PMR. Women were more likely to have PG as the method of induction in countries with a lower PMR as compared to the non-PG approach. In total, 149/368 (40.5%) women underwent a CS after induction of labor. The incidence in the two groups of induction was similar: 62/153 (40.5%) in the PG group and 87/215 (40.5%) in the no PG group. Table 2 outlines the CS rate by method of induction and the variables of interest that were found to be significantly associated with an unplanned CS following induction of labor. The method of induction of labor had no effect on the rate of CS for both twins. Nulliparous women and women over the age of 30 years were more likely to be unsuccessful with planned induction of labor and require an unplanned CS. In addition, a CS was more likely to occur when twin B presentation was non-cephalic and in countries where the national PMR is ≥ 10/1000. Using multiple regression models, parity, maternal age, presentation of twin B and PMR were found to be independently associated with an unplanned CS following induction of twin pregnancies (Table 3). Table 4 outline other neonatal and maternal outcomes, as well as time intervals in the induction and delivery process, for the two groups who underwent induction. The two groups were similar in the incidence of the maternal and neonatal composite outcomes as defined by the protocol in the TBS [1]. The breakdowns of the individual maternal and neonatal outcomes as well as maternal and fetal reasons for cesarean section by therapeutic group are shown in tables Additional file 1: Table S1 and Additional file 2: Table S2, respectively.

**Table 1:** Baseline characteristics by method of induction

Baseline characteristic n (row per cent)	Prostaglandin *N* = 153	No prostaglandin *N* = 215	*p*-value
Maternal age (years)	30.8 ± 6.2	30.1 ± 6.1	0.42
<30	71 (39.4%)	109 (60.6%)	0.42
≥30	82 (43.6%)	106 (56.4%)	
Parity			
0	83 (46.4%)	96 (53.6%)	0.07
≥1	70 (37.0%)	119 (63.0%)	
Gestational age at randomization (weeks)	35.1 ± 1.9	35.0 ± 1.9	0.88
32^0^-33^6^ weeks	55 (43.7%)	71 (56.3%)	
34^0^-36^6^ weeks	61 (38.9%)	96 (61.1%)	0.66
37^0^-38^6^ weeks	37 (43.5%)	48 (56.5%)	
Chorionicity on ultrasound			
Dichorionic diamniotic	117 (43.2%)	154 (56.8%)	0.18
Monochorionic diamniotic	29 (42.0%)	40 (58.0%)	
Unknown	7 (25.0%)	21 (75.0%)	
Birth weight of first twin	2291.9 ± 402.6	2301.9 ± 430.0	0.82
Birth weight of second twin	2276.9 ± 449.7	2322.8 ± 417.2	0.31
National perinatal mortality rate			
<10/1000	116 (45.7%)	138 (54.3%)	
≥10/1000	37 (32.5%)	77 (67.5%)	0.02

Data are mean ± SD

**Table 2:** Cesarean delivery by method of induction and covariables

n (row per cent)	Vaginal delivery for both twins *N* = 219	At least one twin delivered by CS *N* = 149	*p*-value Odds ratio (95% CI)
Method of induction			0.99
PG	91 (59.5%)	62 (40.5%)	1.0 (0.65, 1.5)
No PG	128 (59.5%)	87 (40.5%)	1
Maternal age (years)			0.01
<30	119 (66.1%)	61 (33.9%)	1
≥30	100 (53.2%)	88 (46.8%)	1.7 (1.1, 2.6)
Parity			<0.0001
0	83 (46.4%)	96 (53.6%)	1
≥1	136 (72.0%)	53 (28.0%)	0.33 (0.22, 0.52)
Chorionicity on ultrasound			0.73
Dichorionic	164 (60.5%)	107 (39.5%)	0.75 (0.35, 1.6)
cMonochorionic	40 (58.0%)	29 (42.0%)	0.84 (0.36, 2.0)
Unknown	15 (53.6%)	13 (46.4%)	1
National perinatal mortality rate			0.0032
<10/1000	164 (64.6%)	90 (35.3%)	1
≥10/1000	55 (48.3%)	59 (51.7%)	2.0 (1.2, 3.1)
Presentation of twin B at delivery			0.0012
Cephalic	162 (65.3%)	86 (34.7%)	0.48 (0.31, 0.75)
Non-cephalic	57 (47.5%)	63 (52.5%)	1
Gestational age at delivery			0.93
32^0^-36^6^ weeks	64 (59.3%)	44 (40.7%)	0.86 (0.31, 2.4)
37^0^-38^6^ weeks	145 (59.9%)	97 (40.1%)	0.84 (0.32, 2.2)
39+ weeks	10 (55.6%)	8 (44.4%)	1

**Table 3:** Results of the multivariable logistic regression of cesarean delivery

Variables in the final model		Variables not in the final model	
Variable	*p*-value Odds ratio (95% CI)	Variable	*p*-value^a^ Odds ratio (95% CI) ^a^
Maternal age (years)	0.001	Method of induction	0.61
<30	1	PG	0.88 (0.55, 1.4)
≥30	2.2 (1.4, 3.6)	No PG	1
Parity	<0.0001	Gestational age at randomization	0.73
0	1	32^0^-33^6^ weeks	0.89 (0.47, 1.7)
≥1	0.21 (0.13, 0.35)	34^0^-36^6^ weeks	1.1 (0.61, 2.0)
		37^0^-38^6^ weeks	1
National perinatal mortality rate	<0.0001	Chorionicity on ultrasound	0.55
<10/1000	1	Dichorionic	0.70 (0.30, 1.7)
≥10/1000	3.3 (2.0, 5.6)	Monochorionic	0.92 (0.35, 2.4)
		Unknown	1
Presentation of twin B at delivery	0.0016		
Cephalic	0.46 (0.28, 0.74)		
Non-cephalic	1		

^a^Controlling for variables in the final model

**Table 4:** Other outcome variables by method of induction

Outcome n (column per cent)	Prostaglandin *N* = 153	No prostaglandin *N* = 215	*p*-value Odds ratio (95% CI)^a^
Maternal outcome^b^			
No	137 (89.5%)	189 (87.9%)	0.63
Yes	16 (10.5%)	26 (12.1%)	0.85 (0.44, 1.6)
Outcome N, mean ± SD	Prostaglandin	No prostaglandin	*p*-value
Active labor to dilation (hours)	104, 4.87 ± 4.46	142, 4.91 ± 3.05	0.94
Active labor to delivery (hours)	132, 6.52 ± 5.49	187, 6.6 ± 4.91	0.88
Delivery to discharge (hours)	153, 89.0 ± 54.5	215, 86.7 ± 44.6	0.67
Outcome n (column per cent)	Prostaglandin *N* = 304	No prostaglandin *N* = 428	*p*-value Odds ratio (95% CI)^a^
Neonatal outcome^c^			
No	298 (98.0%)	418 (97.7%)	0.74
Yes	6 (2.0%)	10 (2.3%)	0.86 (0.37, 2.0)

^a^Odds ratio for outcome: prostaglandin vs no-prostaglandin

^b^Composite of maternal death or serious maternal morbidity before 28 days post-partum as defined by the protocol in the TBS [1]

^c^Composite of fetal or neonatal mortality or serious neonatal morbidity as defined by the protocol in the TBS [1]

## Discussion

In this secondary analysis, we were able to demonstrate that the method of induction of labor in twins had no effect on the rate of unplanned CS. In addition, we showed that neither approach contributed to an adverse outcome for either the woman or her infant. However, using either method of induction the rate of an unplanned CS is higher than what has been reported in singleton pregnancies [13–17]. Furthermore, while induction in whom cervical ripening is not required (no PG group) is associated with a lower rate of CS in the case of singleton pregnancies [18, 19], we did not find the same result in twin pregnancies. We postulate that there may be a different mechanism associated with labor and delivery in twins or that the primary higher risk for CS in twins masks this difference [20, 21]. Since CS rate was similar in both groups, we feel this study indicates that all twins in whom induction of labor is indicated should be informed of a significant risk of unplanned CS, regardless of intervention. Late maternal age and nulliparity remained strongly associated with an unplanned CS following induction of labor, as has been shown in many studies [22–24]. On the other hand, the finding that CS occurs more commonly following induction in countries where the PMR is ≥ 10/1000, was not reported previously. It is possible that in low-income countries and in low-resource centers (as a surrogate for higher PMR) physicians are more comfortable with the decision for a controlled CS than with a potentially long and uncontrolled VD in this setting. In the same manner, countries with higher PMR are usually the ones with less economic resources, so it is not surprising that PG (usually a more expensive drug than oxytocin or nearly costless amniotomy) was used less often in these countries. While induction with PG has been reported to be associated with adverse events such as fetal heart rate abnormalities or even uterine rupture [25], we did not find this association or other fetal or maternal adverse outcomes in our trial. Although not powered to assess rare maternal adverse outcomes, this study suggests that both approach of induction of labor, PG or no PG, are safe in twin pregnancies.

Our study’s strength lies in its size (82 participating centers in 23 countries), and a high rate of follow-up. The main limitation of this study is the fact it is a secondary analysis. The method of induction of labor was not randomized and baseline cervical exam, bishop score, the type of PG used and dosing intervals were not available as one of the variables in the analysis between the two groups of induction. Lastly, this study assessed the pharmacological methods of induction (e.g., PG or oxytocin) and is lacking the use of mechanical induction in twin (e.g., single or double balloon catheter) and its comparison with pharmacological methods. Bush et al [11], in a similar study, compared intra-vaginal misoprostol to oxytocin for the induction of labor in twin gestations. They reported a shorter length of induction to delivery and a trend toward a lower CS rate (16.9 vs. 31.6%, *p* = 0.06) in the oxytocin-only group. They too concluded that both PG and oxytocin appear to be safe and efficacious for use in induction of labor in twins. Huber et al [26] and Okby et al [21] analyzed twin induction using PG followed by amniotomy/oxytocin as an induction method. They found CS rate with induction of labor in twin pregnancy to be similar to our study (43.7 and 31.2%, respectively). Taylor et al [3] and Suzuki et al [7], in a study in which a variety of methods of induction were used, found CS rate to be lower than our study (19 and 18%, respectively). In their studies however only induced women for the indication of advanced gestational age where included, whereas our study other indications for induction were included which may explain our higher CS rate. In a recent study, de Castro et al [27] found induction of labor with Foley catheter to be an independent risk factor for having CS. In their retrospective study of 883 women with twins who underwent a trial of labor, other methods of induction were not assessed and information regarding neonatal mortality and morbidity was missing. As in our study, others have shown that both PG and oxytocin are safe induction agents in twin pregnancy and are not associated with higher rates of hyperstimulation or adverse neonatal or maternal outcomes compared to induction in singleton [3, 11, 12, 28].

## Conclusion

Both methods of induction, PG or no PG, are associated with the same high rate of unplanned CS, but are safe for use in induction of labor in twins. Further studies are needed to determine the effectiveness and safety of various induction methods in twin pregnancy.

## Additional files

Additional file 1: Table S1. Adverse maternal or neonatal outcomes by method of induction. (RTF 86 kb)
Additional file 2: Table S2. Maternal and fetal reasons for cesarean section by therapeutic group. (RTF 52 kb)

## Abbreviations

CS: Cesarean section
PG: Prostaglandins
PMR: Perinatal mortality rate
RCT: Randomized controlled trial
TBS: Twin birth study
VD: Vaginal delivery
