# Supplementary material for: The effect of induction method in twin pregnancies: a secondary analysis for the twin birth study
Source: BMC Pregnancy Childbirth. 2017 Jan 6;17:9. doi: 10.1186/s12884-016-1201-8 (PMC5217445; doi:10.1186/s12884-016-1201-8)
Supplement: Additional file 1: Table S1. — Adverse maternal or neonatal outcomes by method of induction. (RTF 86 kb) [file 12884_2016_1201_MOESM1_ESM.rtf]

Table Supplement 1. Adverse maternal or neonatal outcomes by method of induction
Maternal morbidity 
n (column per cent)	Prostaglandin
N=153	No prostaglandin
N=215	
Hemorrhage§ v
Missing	15 (10.0%)
2	20 (9.4%)
1	
Laparotomy v	0 (0.0%)	0 (0.0%)	
Genital tract injury	0 (0.0%)	0 (0.0%)	
Perineal injury§	36 (23.7%)	58 (27.0%)	
Thromboembolism requiring anticoagulant therapy v	
0 (0.0%)	
1 (0.5%)	
Maternal infection§ v	0 (0.0%)	5 (2.3%)	
Wound infection§	5 (3.3%)	1 (0.5%)	
Wound dehiscence or wound breakdown v	1 (0.7%)	2 (0.9%)	
Major medical life threatening illness§	0 (0.0%)	0 (0.0%)	
Neonatal morbidity 
n (column per cent)	Prostaglandin
N=301 	No prostaglandin
N=425 	
Birth trauma†	0 (0.0%)	2 (0.5%)	
Apgar at 5 minutes < 4†	1 (0.3%)	2 (0.5%)	
Abnormal level of consciousness†
Coma
Hyperalert, drowsy, or lethargic	
0 (0.0%)
1 (0.3%)	
0 (0.0%)
1 (0.2%)	
≥ 2 neonatal seizures within 72 hours of age†	1 (0.3%)	1 (0.2%)	
≥ 24 hours of ventilation via endotracheal tube initiated within 72 hours of age†	
0 (0.0%)	
2 (0.5%)	
Infection within 72 hours of age†	0 (0.0%)	0 (0.0%)	
Necrotising enterocolitis†	1 (0.3%)	0 (0.0%)	
Intraventricular hemorrhage†	0 (0.0%)	0 (0.0%)	
Cystic periventricular leukomalacia†	0 (0.0%)	0 (0.0%)	
§ More than one response may apply.
v Components of the maternal composite outcome as defined by the protocol in the Twin Birth Study [1].
† Components of the neonatal composite outcome as defined by the protocol in the Twin Birth Study [1].
