# Supplementary material for: The effect of induction method in twin pregnancies: a secondary analysis for the twin birth study
Source: BMC Pregnancy Childbirth. 2017 Jan 6;17:9. doi: 10.1186/s12884-016-1201-8 (PMC5217445; doi:10.1186/s12884-016-1201-8)
Supplement: Additional file 2: Table S2. — Maternal and fetal reasons for cesarean section by therapeutic group. (RTF 52 kb) [file 12884_2016_1201_MOESM2_ESM.rtf]

Table Supplement 2. Maternal and fetal reasons for cesarean section by therapeutic group.
Reasons for cesarean*	Prostaglandins
(N=62)	No prostaglandins
(N=87)	
Maternal
 Failed induction of labor
 Maternal complication
 Antepartum hemorrhage
 Patient request
 Gestational age reached 375/7-386/7 wk
 Hypertension/pre-eclampsia
 Maternal discomfort
 Other reason
Fetal
 Fetal compromise
 Fetal malpresentation
 Failure to progress
 Cord presentation or prolapse
 Failed forceps or vacuum
 Failed external cephalic/internal podalic version
 Fetal death
 Other reason	
34
6
0
5
1
2
0
2

14
5
20
1
2
1
0
0	
57
6
2
7
3
1
1
2

21
2
24
1
1
0
0
0	
* More than one response may apply.
